# Two DELLA-interacting proteins bHLH48 and bHLH60 regulate flowering under long-day conditions in *Arabidopsis thaliana*

**DOI:** 10.1093/jxb/erx143

**Published:** 2017-06-07

**Authors:** Yang Li, Houping Wang, Xiaoli Li, Gang Liang, Diqiu Yu

**Affiliations:** 1 Key Laboratory of Tropical Plant Resources and Sustainable Use, Xishuangbanna Tropical Botanical Garden, Kunming, Yunnan, China; 2 University of Chinese Academy of Sciences, Beijing, China

**Keywords:** *Arabidopsis thaliana*, bHLH48, bHLH60, DELLA, flowering, GA

## Abstract

Gibberellin (GA) regulates many developmental transitions in the plant life cycle. Although great progress has been made, the GA signaling pathways have not been fully elucidated. Identifying and characterizing new targets of DELLA proteins is an effective approach to reveal the complicated GA signaling networks. In this study, two novel DELLA-interacting transcription factors, bHLH48 and bHLH60, were identified. Their overexpression caused plants to flower early under long-day conditions, whereas their functional repression resulted in the opposite result. The constitutive expression of *bHLH48* and *bHLH60* upregulated the transcription of the *FLOWERING LOCUS T* (*FT*) gene. Chromatin immunoprecipitation experiments confirmed that bHLH48 bound to the promoter of *FT* and that GA promoted the DNA-binding activity of bHLH48. Genetic analyses indicated that the early flowering phenotype of plants overexpressing *bHLH48* and *bHLH60* depended on *FT* and that the overexpression of *bHLH48* and *bHLH60* could rescue the late-flowering phenotypes of *RGL1* overexpressing plants. Transient expression assays suggested that RGL1 inhibited the transcription activation ability of bHLH48 and bHLH60. Taken together, this study confirmed that bHLH48 and bHLH60 positively regulate GA-mediated flowering.

## Teaser

bHLH48 and bHLH60 regulate GA-mediated flowering by activating the expression of *FT* under long-day conditions.

## Introduction

Gibberellin (GA) is essential for many developmental processes throughout the entire life cycle of plants, including seed germination, hypocotyl elongation and floral transition. Thus, mutant plants that are deficient in GA exhibit altered seed germination, dwarf and late-flowering phenotypes (Sun, 2008; Achard and Genschik, 2009).

The current model of GA action proposes that DELLA proteins restrain plant growth, whereas GA promotes growth by overcoming DELLA-mediated growth restraint (Harberd, 2003; Achard and Genschik, 2009; Davière and Achard, 2013). GA is perceived by its receptor, GIBBERELLIN INSENSITIVE DWARF1 (GID1a, b and c) (Ueguchi-Tanaka *et al.*, 2005; Nakajima *et al.*, 2006; Griffiths *et al.*, 2006), which stimulates the formation of the GA-GID1-DELLA complex. This ultimately leads to degradation of the transcriptional regulator DELLA proteins, including GA INSENSITIVE (GAI), REPRESSOR OF GA1-3 1 (RGA), RGA-LIKE1 (RGL1), RGL2, and RGL3 (Murase *et al.*, 2008; Harberd *et al.*, 2009; Sun, 2010). DELLA proteins lack a canonical DNA-binding domain and exert their molecular functions through interaction with diverse classes of regulatory proteins, such as PHYTOCHROME INTERACTING FACTORS (PIFs) (de Lucas *et al.*, 2008; Feng *et al.*, 2008), BRASSINAZOLE RESISTANT1 (BZR1) (Bai *et al.*, 2012; Gallego-Bartolomé *et al.*, 2012), JASMONATE INSENSITIVE1 (JIN1/MYC2) (Hong *et al.*, 2012; Wild *et al.*, 2012), and JASMONATE ZIM-DOMAIN (JAZ) (Hou *et al.*, 2010).

GA has been known to regulate flowering. The *ga1-3* mutant, which has impaired GA biosynthesis, fails to flower in short-day (SD) conditions but shows a relatively weak late flowering phenotype under long-day (LD) conditions (Wilson *et al.*, 1992), suggesting that GA is required for floral transition under SD conditions. Our recent study confirms that DELLA proteins GAI and RGL1 interact with WRKY12 and WRKY13 and that this interaction interferes with the transcriptional activity of WRKY12 and WRKY13 with their downstream gene *FRUITFULL* (*FUL*). GA application assays indicate that WRKY12 and WRKY13 are involved in controlling the timing of GA-mediated flowering under SD conditions (Li *et al.*, 2016). Accumulating evidence strongly indicates that GA also plays an important role in regulating flowering under LD conditions (Griffiths *et al.*, 2006; Willige *et al.*, 2007). As the main contributor towards the LD flowering transition, *FT* is induced under LD conditions and its upregulation does not occur in the *ga1-3* mutant (Hisamatsu and King, 2008). When the expression of *GIBBERELLIN 2 OXIDASE 7* (*GA2ox7*), the product of which can catabolize active GAs, was driven by *cauliflower mosaic virus (CaMV) 35S*, *SUCROSE TRANSPORTER 2* (*SUC2*) and *KNOTTED-LIKE FROM ARABIDOPSIS THALIANA 1 (KNAT1*) promoters, the depletion of GAs in the vascular tissue *(SUC2*) or shoot apical meristem (*KNAT1*) caused delayed flowering and reduced transcript abundance of *FT* under LD conditions (Porri *et al.*, 2012). Tissue-specific misexpression of DELLA genes led to delayed flowering under LD conditions (Galvão *et al.*, 2012), implying that DELLA proteins play pivotal roles in GA-mediated flowering under LD conditions. This evidence suggests that GA is required for the increase of *FT* in vascular tissue under LD conditions. However, the molecular mechanism of *FT* upregulation mediated by GA under LD conditions remains unclear. A recent study confirmed that DELLAs interact with and suppress SQUAMOSA PROMOTER BINDING-LIKE (SPL) proteins, which can activate the transcription of *FT* by binding directly to the *FT* promoter (Kim *et al.*, 2012) and indirectly affect the miR172-*AP2*-*FT* signaling cascade (Yu *et al.*, 2012). However, *Pro35S:MIR156* plants, in which those *SPL* genes are silenced, still flower early after GA application, indicating that other flowering-associated components involved in GA-mediated flowering likely exist (Yu *et al.*, 2012). In *co-2* mutant plants, exogenous application of GA3 was almost unable to induce *FT* expression, suggesting that GA-induced expression of *FT* under LD conditions is dependent on *CONSTANS* (*CO*) (Wang *et al.*, 2016). Mechanistic investigation reveals that DELLA proteins physically interact with and inhibit CO, resulting in the reduction of *FT* expression (Wang *et al.*, 2016). However, the flowering time of *gai-t6 rga-t2 rgl1-1 rgl2-1 co-2* mutant (*dellap co-2*) is still earlier than that of the *co-2* single mutant plants, implying that other DELLA-repressed flowering-related targets may exist.

Here, two DELLA interacting proteins, bHLH48 and bHLH60, were identified. Overexpression of *bHLH48* and *bHLH60* caused early flowering under LD conditions by directly activating the transcription of *FT*. RGL1 interacted with bHLH48 and bHLH60 and this interaction could reverse the activation ability of bHLH48 and bHLH60 in regards to *FT*. This work reveals that bHLH48 and bHLH60 are two novel transcription factors involved in GA-mediated LD flowering.

## Materials and methods

### Plant materials

*Arabidopsis thaliana* ecotype Columbia (Col-0) was used for all experiments. *ft-11* was kindly provided by Prof. Ligeng Ma (Capital Normal University, China). *bhlh48-1* (SALK_092968) and *bhlh48-2* (SALK_013047) mutants were obtained from the Arabidopsis Biological Resource Center. The T-DNA insertions were confirmed using PCR with a T-DNA primer and gene-specific primers (see Supplementary Table S1 at *JXB* online). Seeds were surface-sterilized with 20% bleach and washed three times with sterile water. Sterilized seeds were suspended in 0.1% agarose and plated on Murashige and Skoog (MS) medium. After vernalization for 2 d in the dark at 4 °C, the plates were transferred to the culture room at 22 °C under LD conditions (16-h light/8-h dark) or SD conditions (8-h light/16-h dark). 7-day-old seedlings were planted in soil maintained in an artificial growth chamber at 22 °C under LD conditions or SD conditions. For the transgenic plants, homozygous T3 generation seeds were used unless specifically indicated.

### Expression analysis

One microgram of total RNA extracted from Arabidopsis seedlings using the Trizol reagent (Invitrogen) was used for oligo(dT)18-primed cDNA synthesis, according to the reverse transcription protocol (Fermentas). The resulting cDNA was subjected to qRT-PCR using a SYBR Premix Ex Taq kit (Takara) on a Roche LightCycler 480 real-time PCR machine, according to the manufacturer’s instructions. The results were normalized to the expression level of *ACT2*. Data presented are mean values of three biological repeats with SD. The qRT-PCR primers used are listed in Supplementary Table S1.

### Construction of plasmids

To generate the overexpressing transgenic plants, an HA tag was fused with the N-terminal of *bHLH48* or *bHLH60* and cloned into vector pOCA30 in the sense orientation behind the CaMV 35S promoter. To construct *35S:RGL1*, the full-length cDNA of *RGL1* was cloned into the vector pOCA30 in the sense orientation behind the CaMV 35S promoter. For the *35S:bHLH48-EAR* plasmids, the full-length cDNA of *bHLH48* was fused with a 12-amino acid repression domain (LDLDLELRLGFA). All of the constructs were then transformed into *Agrobacterium tumefaciens* strain GV3101. Arabidopsis transformation was performed by the floral dip method. Transgenic plants were selected using 50 mg/mL kanamycin. The primers used are listed in Supplementary Table S1.

### GUS reporter analysis

To generate GUS reporter transgenic plants, 3.4 kb and 2.5 kb upstream sequences from the translation start site of *bHLH48* and *bHLH60* were separately amplified from genomic DNA and cloned into vector pOCA28. Transgenic plants were subjected to GUS staining as described previously (He *et al*., 2014). The primers used are listed in Supplementary Table S1.

### Yeast two-hybrid assay

*RGL1* with a deletion of 159 amino acids in the N-terminal was cloned into vector pGBKT7 and then transformed into the yeast strain Y2HGold (Clontech). The cDNA library was obtained from Clontech (catalog number 630487). Two-Hybrid screening was performed via the mating protocol described in Clontech’s Matchmaker^TM^ Gold Yeast Two-Hybrid user manual. To confirm the interactions, the full-length coding sequences of bHLH48 and bHLH60 proteins were cloned into vector pGADT7. Growth was determined as described in the Yeast Two-Hybrid System User Manual (Clontech). The primers used for vector construction are listed in Supplementary Table S1.

### BiFC assay

Full-length coding sequences of *bHLH48* and *bHLH60* were cloned into binary vectors containing nYFP, while the full-length coding sequences of *RGL1* were cloned into the vector containing cYFP. Agrobacterium strains transformed with the indicated nYFP or cYFP vectors were incubated, harvested and resuspended in infiltration buffer, comprising 0.2 mM acetosyringone, 10 mM MgCl_2_ and 10 mM MES at pH 5.6, to identical concentrations (A600=0.5). Equal volumes of an Agrobacterium culture containing nYFP (A600=0.5) and cYFP (A600=0.5) were mixed before infiltration into *Nicotiana benthamiana* leaves. After infiltration, plants were incubated at 24 °C for 48 h before observation.

### Co-Immunoprecipitation (Co-IP) assay

For Co-IP assays, the full-length coding sequence of *bHLH48*, *GFP* or *RGL1* was amplified and cloned into tagging vectors behind the MYC or Flag tag in the sense orientation behind the CaMV 35S promoter. The constructs were transformed into *A. tumefaciens*. Myc-bHLH48 (or Myc-GFP) and Flag-RGL1 were transiently co-expressed in tobacco leaves. All infected leaves were treated with 10 mM MG132 and 20 mM paclobutrazol, a GA biosynthesis inhibitor, 40 h after infiltration. After 8 h, those leaves were harvested and used for protein extraction. Finally, Flag-RGL1 was immunoprecipitated using Flag antibody and the co-immunoprecipitated proteins were detected using Myc antibody (Sigma-Aldrich).

### Gene editing

Gene editing was conducted as described previously (Liang *et al.*, 2016). Briefly, one sgRNA containing one target sequence was fused with the AtU3b promoter and inserted into the multiple clone site in the pMH-SA vector. The target sequence is shown in Supplementary Fig. S1. Homozygous mutants were isolated from T2 generation plants by sequencing. The primers used are listed in Supplementary Table S1.

### Transient expression assays

The *LUCIFERASE* (*LUC*) gene under control of the 2.6 kb *FT* promoter was used as a reporter (*ProFT:LUC*) and the 35S promoter-driven *RENILLALUCIFERASE* (*REN*) was the internal control. *bHLH48*, *bHLH60*, and *GFP* were fused downstream of the 35S promoter in vector pGreenII 62-SK to serve as effectors; *35S:bHLH48*, *35S:bHLH60* and *35S:GFP*. All primers used are listed in Supplementary Table S1. Arabidopsis mesophyll protoplasts were prepared, transfected and cultured as previously described (Yoo *et al*., 2007). Firefly and control REN activities were assayed from leaf extracts using the Dual-Glo Luciferase Assay System (Promega) and quantified using a GloMax 96 Microplate Luminometer (Promega). Data presented are mean values of three biological repeats.

## Results

### RGL1 interacts with bHLH48 and bHLH60

**Fig. 1.**
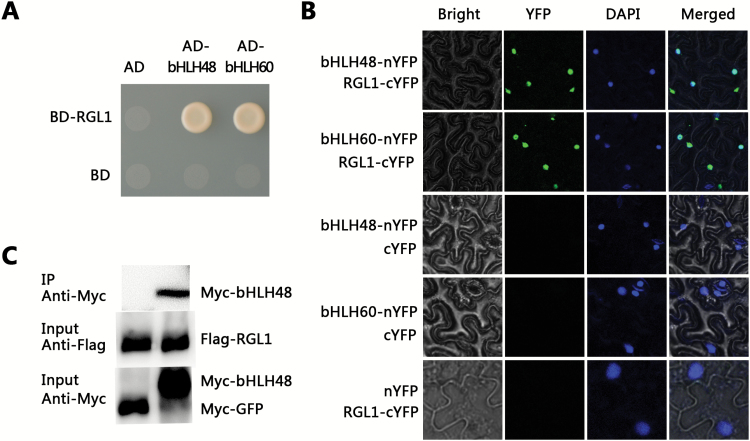
RGL1 interacts with bHLH48 and bHLH60. (A) Yeast two-hybrid assays. Interaction was indicated by the ability of cells to grow on synthetic dropout medium lacking Leu/Trp/His/Ade. Truncated RGL1 and full-length bHLH48 or bHLH60 were cloned into pGBKT7 (shown as BD) and pGADT7 (shown as AD), respectively. (B) BiFC analysis. Fluorescence was observed in nuclear compartments of *N. benthamiana* leaf epidermal cells. DAPI, 4’,6-diamidino-2-phenylindole. (C) Co-IP assay. Total protein was immunoprecipitated using Flag antibody and co-immunoprecipitated protein was then detected using Myc antibody.

To identify novel components involved in GA signaling pathways, the yeast-two-hybrid system was employed to screen for DELLA interacting proteins. Due to the autoactivation of RGL1 in yeast, RGL1 with a deletion of 159 amino acids in its N-terminal was used as bait (BD-RGL1). PIF4 was used as a positive control (de Lucas *et al.*, 2008). After screening, the bHLH transcription factor bHLH48 (At2g42300) was identified as a candidate RGL1 interacting protein. Transciption factor bHLH48 belongs to bHLH subgroup XII (Heim *et al.*, 2003). Construction of a phylogenetic tree revealed that bHLH60 (At3g57800) is a close homolog of bHLH48 with 68% identity (Supplementary Fig. S2). Further experiments revealed that both bHLH48 and bHLH60 could interact with RGL1 in yeast (Fig. 1A). To further confirm the interaction between bHLH48 and bHLH60 with RGL1 *in planta*, a bimolecular fluorescence complementation (BiFC) assay was used. Fusion proteins were generated, with bHLH48 and bHLH60 fused with the N-terminal fragment of yellow fluorescent protein (nYFP) and RGL1 fused to the C-terminal fragment (cYFP). When bHLH48-nYFP or bHLH60-nYFP were transiently co-expressed with RGL1-cYFP, strong YFP fluorescence was visible in the nucleus of epidermal cells in *N. benthamiana* leaves, whereas no YFP fluorescence was detected in the negative controls i.e. bHLH48-nYFP or bHLH60-nYFP co-expressed with cYFP or RGL1-cYFP co-expressed with nYFP (Fig. 1B). The interaction between bHLH48 and RGL1 was further corroborated by co-IP assays in plant cells (Fig. 1C). Taken together, these data suggest that both bHLH48 and bHLH60 could physically interact with RGL1.

### bHLH48 and bHLH60 positively regulate flowering under LD conditions

**Fig. 2.**
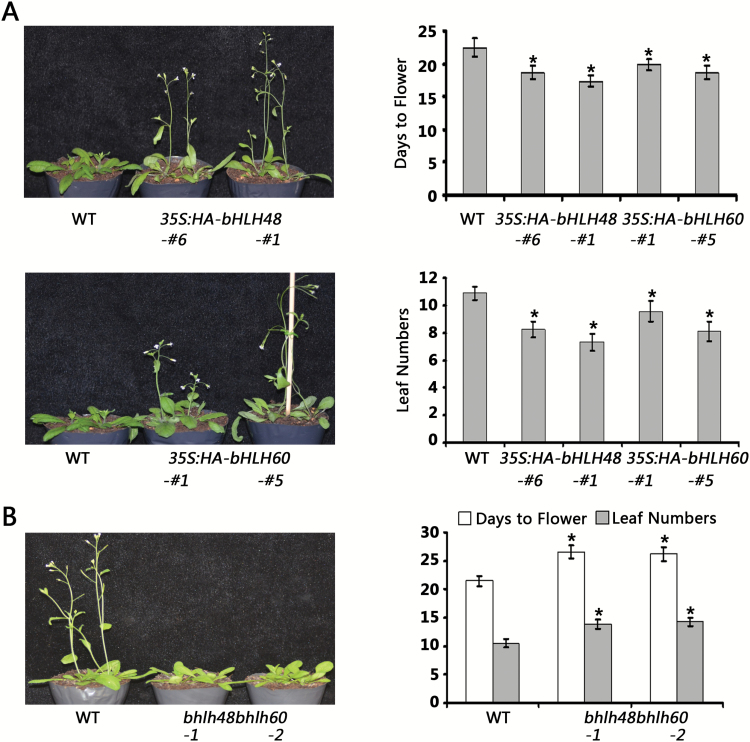
*bHLH48* and *bHLH60* positively regulate flowering under LD conditions. (A) Flowering phenotype in *bHLH48* and *bHLH60* overexpressing plants; 25-day-old plants are shown. The experiment was repeated at least three times, with similar results obtained. (B) Flowering phenotype in *bhlh48bhlh60* double mutants; 28-day-old plants are shown. The experiment was repeated at least three times, with similar results obtained. (A) and (B) The quantitative flowering times were measured as days to flower and the number of rosette leaves at the day floral buds became visible. The mean values (*n*≥20) are shown, with bars indicating standard deviation. Significant differences from the wild-type are indicated by *, *P* < 0.05.

To explore the functions of *bHLH48* and *bHLH60*, overexpressing transgenic plants were generated, *35S:HA-bHLH48* and *35S:HA-bHLH60*. The transcript abundance of the transgenes was examined using quantitative real-time PCR (Supplementary Fig. S3). When grown under LD conditions, the overexpressing transgenic plants flowered significantly earlier than the wild-type plants (Fig. 2A). In contrast, their flowering phenotype was comparable to that of wild-type under SD conditions (Supplementary Fig. S4). To further investigate the functions of *bHLH48* and *bHLH60*, T-DNA insertion lines for *bHLH48 and bHLH60* were obtained from the Arabidopsis Biological Resource Center. Two homozygous T-DNA insertion lines, *bhlh48-1* and *bhlh48-2* (Supplementary Fig. S1), were identified for *bHLH48*. No T-DNA insertion was available for *bHLH60* and consequently two independent mutants, *bhlh60-1 and bhlh60-2*, were constructed using CRISPR/Cas9 DNA editing (Supplementary Fig. S1). These mutants showed no visible difference in flowering time compared with wild-type plants, either grown under LD or SD conditions (Supplementary Fig. S1). Considering the potential functional redundancy between bHLH48 and bHLH60, two double mutants, *bhlh48bhlh60-1* and *bhlh48bhlh60*-*2*, were generated through genetic crossing of *bhlh48-1* with *bhlh60-1* or *bhlh60-2*. Although the double mutants and wild-type plants flowered at the same time under SD conditions (Supplementary Fig. S5), the double mutants showed a statistically significant delay in flowering compared with wild-type plants under LD conditions (Fig. 2B).

A dominant repression strategy (Hiratsu *et al.*, 2003) was also employed to confirm the functions of bHLH48 and bHLH60. A 12 amino acid ERF-associated amphiphilic repression (EAR) motif, which serves as a strong repressor domain, was fused in frame with the 3’ end of bHLH48 (Supplementary Fig. S6) to generate a dominant repression line, *35S:bHLH48-EAR*. When grown under LD conditions, *35S:bHLH48-EAR* plants showed a dramatically delayed flowering phenotype compared with wild-type plants (Supplementary Fig. S6). Taken together, these data suggest that bHLH48 and bHLH60 positively regulate flowering under LD conditions in *A. thaliana.*

### *The early flowering phenotype of overexpression plants depends on* FT

**Fig. 3.**
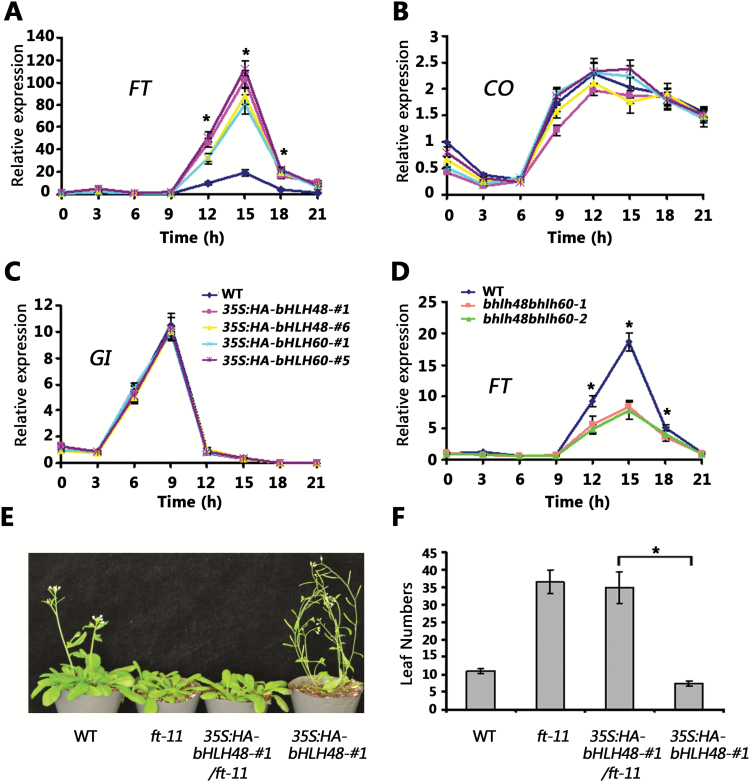
*FT* is required for the early flowering phenotype of overexpression plants. (A–C) Daily expression patterns of *FT*, *CO* and *GI* in 8-day-old overexpressing transgenic plants under LD conditions. (D) Daily expression patterns of *FT* in 8-day-old *bhlh48bhlh60* double mutants. (A-D) The data represent means ± standard deviation of three biological repeats. Significant differences from the wild-type are indicated by *, *P* < 0.05. (E–F) Flowering phenotype of *35S:HAbHLH48-#1*, *35S:HAbHLH48-#1/ft-11*, *ft-11* and wild-type plants. Plants were grown under LD conditions for 30 days when the pictures were taken. The quantitative flowering times were measured as the number of rosette leaves at the day floral buds became visible. Mean values (*n*≥20) are shown, with bars indicating standard deviation. Significant differences between two samples are indicated by *, *P* < 0.05.

To determine the potential causes of the early flowering phenotype of the overexpressors, the expression levels of the photoperiodic pathway genes *FT*, *CO* and *GI* were examined. RNA levels were measured every 3 hours over a 24-hour cycle under LD conditions. The abundance of *FT* mRNA was particularly high in the *bHLH48* and *bHLH60* overexpressing plants, however the circadian rhythm of *FT* was not affected (Fig. 3A). By contrast, transcript levels of *CO* and *GI* were not significantly affected (Fig. 3B, C). Further examination found that the transcript levels of *FT* in the *bhlh48bhlh60* double mutants were dramatically reduced when compared with wild-type plants (Fig. 3D). These results suggest that elevated *FT* levels might be required for the early flowering phenotype in the overexpressing lines. To confirm this hypothesis, the *ft-11* mutation (Yang *et al.*, 2012) was introduced into *35S:HA-bHLH48* plants. As expected, the *35S:HA-bHLH48/ft-11* plants showed an obvious late-flowering phenotype under LD conditions, which was similar to that of *ft-11* (Fig. 3E, F). This result revealed that the early flowering phenotype of *35S:HA-bHLH48* is dependent on upregulation of the *FT* gene.

### bHLH48 directly activates the transcription of the *FT* gene by associating with its chromatin regions

**Fig. 4.**
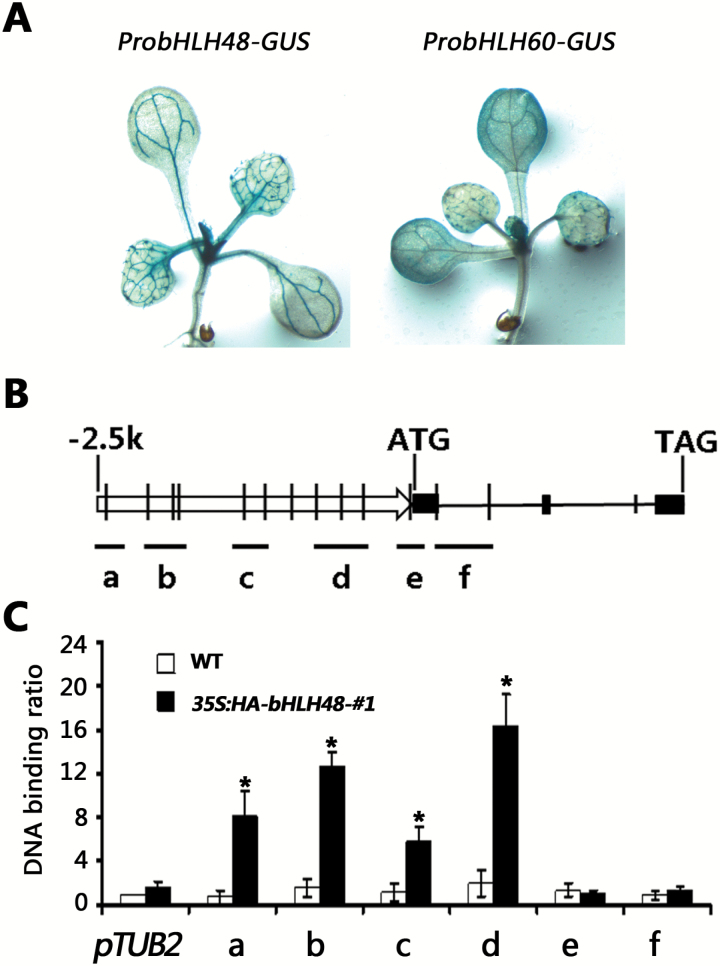
bHLH48 directly binds to the promoter of *FT*. (A) Expression patterns of *bHLH48* and *bHLH60*. Plants were grown under LD conditions for 8 days and the whole plants used for GUS staining. (B) A diagram depicting the putative promoter (arrow), exons (black boxes) and introns (black lines) of the *FT* gene. The vertical black lines indicate positions of E-boxes (5’-CANNTG-3’). The black lines from a–f depict the DNA regions that were amplified or not amplified by ChIP-PCR using the indicated primer sets. (C) ChIP-PCR results. Chromatin fragments were prepared from 8-day-old wild-type or *35S:HA-bHLH48-#1* seedlings and immunoprecipitated using the antibody against HA. Quantitative PCR was used to quantify the enrichment of the *FT* gene promoter. A fragment of the *TUB2* promoter containing an E-box motif was used as a negative control. The binding of the *TUB2* promoter fragment in the wild-type was set to 1 and used to normalize the DNA binding ratio of the *FT* gene promoter. The mean values (*n*=3) are shown, with bars indicating standard deviation. Significant differences from the wild-type are indicated by *, *P* < 0.05.

Considering that *FT* transcript abundance was upregulated in the overexpressing plants and downregulated in the double mutants, it was speculated that *FT* is regulated directly by bHLH48 and bHLH60. If bHLH48 and bHLH60 regulate *FT* directly, their spatial expression patterns should overlap with that of *FT*. To determine the expression patterns of *bHLH48* and *bHLH60*, the putative promoters of *bHLH48* and *bHLH60* were used to drive the GUS reporter gene. *ProbHLH48-GUS* and *ProbHLH60-GUS* transgenic plants were generated. Although these promoters may not contain all *cis*-elements, they were active in the vascular bundle cells (Fig. 4A), where *FT* is expressed (Takada and Goto, 2003), implying a potential link between *bHLH48* and *bHLH60* with *FT*.

The bHLH transcription factors regulate their target genes by association with E-box (5’-CANNTG-3’) *cis*-elements in the promoters of their target genes (Fisher and Goding, 1992). Bioinformatics analysis showed that the putative promoter region of *FT* contains several E-box motifs (Fig. 4B). Considering the high functional redundancy between bHLH48 and bLHL60, only bHLH48 overexpressing plants were chosen as a representative to conduct a ChIP assay. For the ChIP assay, *35S:HA-bHLH48* transgenic plants grown under LD conditions were harvested at Zeitgeber time 16 (ZT16) when *FT* expression is at its peak. A fragment of the *TUB2* promoter containing an E-box motif was used as a negative control. HA-bHLH48 specific enrichments of DNA fragments at different *FT* locations i.e. amplicons a-f, were analyzed using qRT-PCR. As shown in Fig. 4C, bHLH48 was associated with region a, b, c and d of the *FT* promoter, which contains E-box sequences. These data suggested that bHLH48 binds directly to the promoter of the *FT* gene.

### GA promotes the DNA-binding activity of bHLH48 with *FT*

**Fig. 5.**
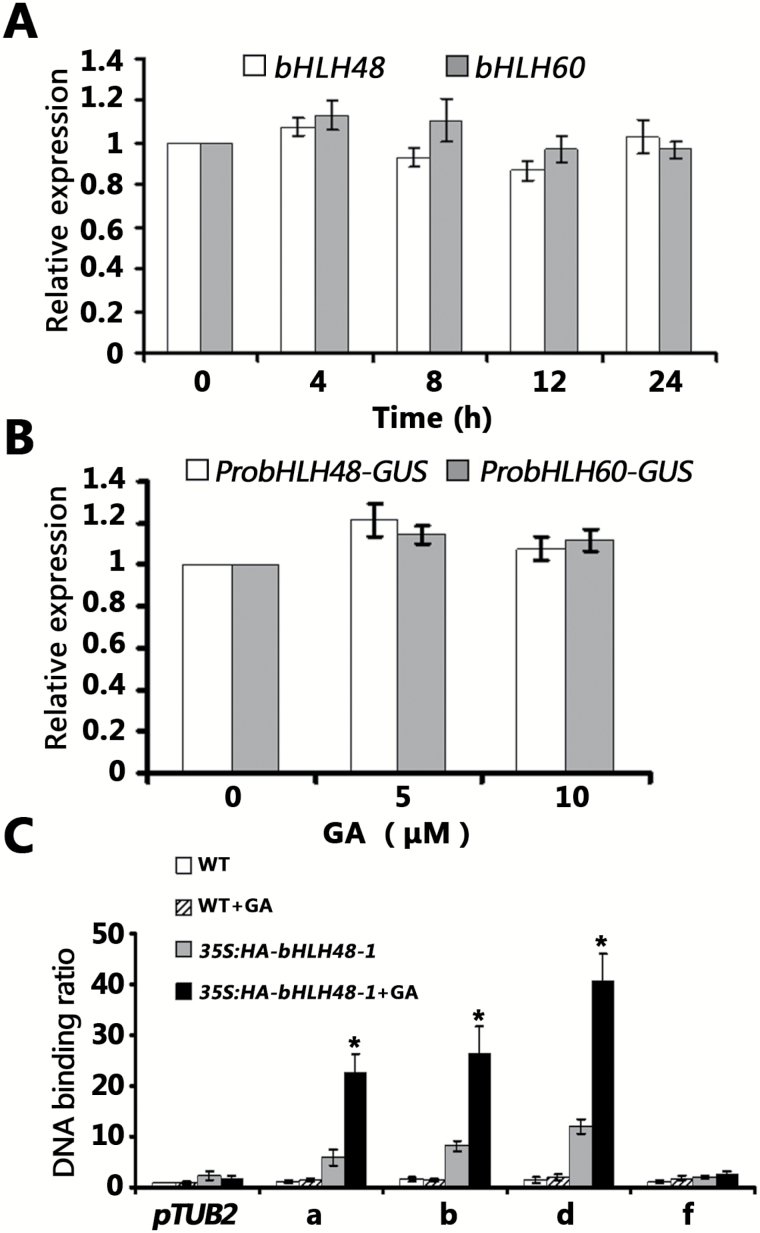
GA promotes the DNA-binding activity of bHLH48 with *FT*. (A) Expression of *bHLH48* and *bHLH60* in response to GAs. The 7-day-old seedlings were treated with 50 µM GA3 for the indicated times. Total RNA was isolated for the following experiments. (B) Relative GUS expression levels of *ProbHLH48-GUS* and *ProbHLH60-GUS* plants on GA application. Seedlings were grown on half MS media containing indicated concentration of GA3 in LD for 8 days. Total RNA was isolated for the following experiments. (C) ChIP-PCR results. Chromatin fragments were prepared from 8-day-old wild-type or *35S:HA-bHLH48-#1* seedlings grown on half MS media containing 0 or 10 µM GA3. Chromatin fragments were immunoprecipitated using the antibody against HA. Quantitative PCR was used to quantify the enrichment of the *FT* promoter. A fragment of the *TUB2* promoter containing an E-box motif was used as a negative control. The binding of the *TUB2* promoter fragment in the wild-type was set to 1 and used to normalize the DNA binding ratio of the *FT* promoter. The mean values (*n*≥20) are shown, with bars indicating standard deviation. Significant differences with the samples lacking GA application are indicated by *, *P* < 0.05.

To further elucidate the roles of bHLH48 and bHLH60 in the regulation of the GA signaling pathway, the expression levels of *bHLH48* and *bHLH60* were examined after exogenous GA application. As shown in Fig. 5A, transcript abundance of both *bHLH48* and *bHLH60* were not affected by exogenous GA application. Similarly, the GUS expression levels of *ProbHLH48-GUS* and *ProbHLH60-GUS* were also not affected by exogenous GA application (Fig. 5B). It was proposed that exogenous GA application could promote the DNA-binding activity of *bHLH48* and *bHLH60* with *FT*. To test this, *bHLH48* overexpressing plants were chosen to conduct a ChIP assay. For the ChIP assay, transgenic plants grown under LD conditions on media with or without GA3 were harvested at ZT16. As shown in Fig. 5C, HA-bHLH48 specific enrichments of DNA fragments on a, b, and d of the *FT* promoter in GA3 application plants, were greater than that in plants without GA3 application. Region f was used as a negative control. Taken together, these results indicate that GA promotes the DNA-binding activity of *bHLH48* with the *FT* promoter.

### RGL1 represses the transactivation ability of bHLH48 and bHLH60

**Fig. 6.**
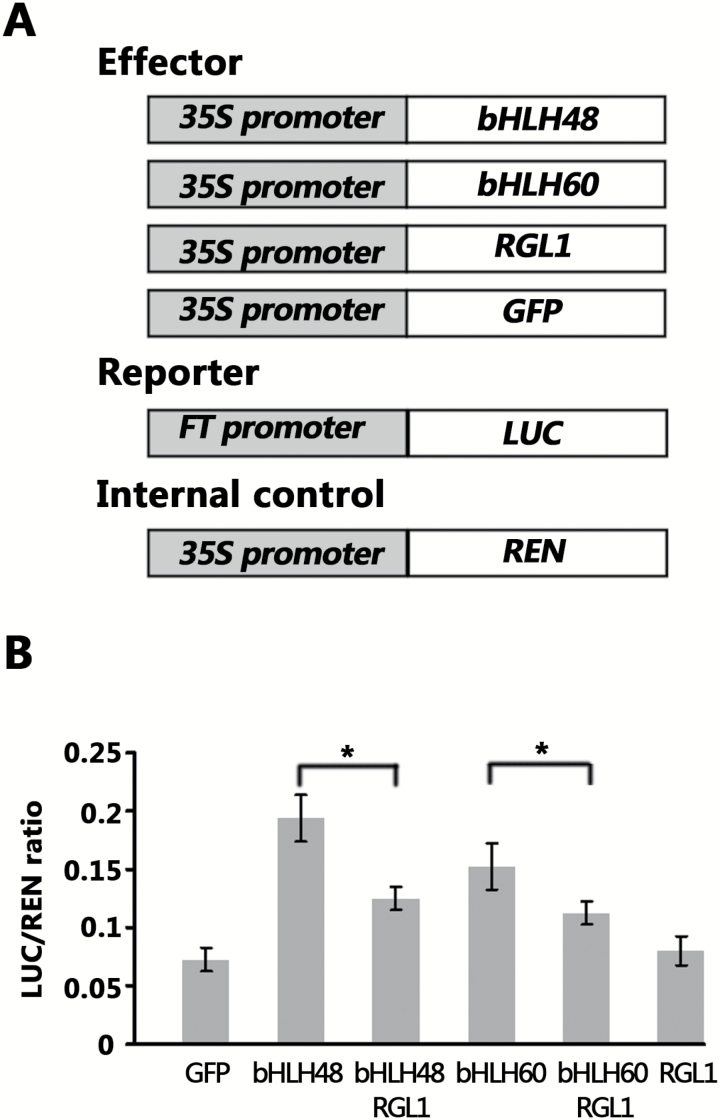
Transactivation inhibition of bHLH48 and bHLH60 by RGL1. (A) Constructs used in transient transactivation assays. Grey bar indicates promoter. White bar indicates gene. (B) RGL1 represses the transactivation ability of bHLH48 and bHLH60. A transient expression assay was performed in Arabidopsis protoplasts. *35S:bHLH48*, *35S:bHLH60*, *35S:RGL1* and *35S:GFP* serve as effectors, *ProFT:LUC* as the reporter and *35S:REN* as the internal control. The mean values (*n*=3) are shown, with bars indicating standard deviation. Significant differences between the indicted two samples are indicated by *, *P* < 0.05.

Previous studies revealed that DELLA proteins can block the DNA-binding capacity and inhibit the transcriptional regulatory activity of target transcription factors (de Lucas *et al.*, 2008; Hou *et al.*, 2010; Gallego-Bartolomé *et al.*, 2012; Resentini *et al*., 2015). Having confirmed the interaction of RGL1 with bHLH48 and bHLH60, we were interested in whether RGL1 affects the transcriptional regulatory activity of bHLH48 and bHLH60. To this end, an Arabidopsis protoplast transient expression assay based on an effector-reporter system was performed (Fig. 6A). In order to serve as effectors, *bHLH48* and *bHLH60* were located downstream of the *35S* promoter in the vector pGreenII 62-SK (*35S:bHLH48* and *35S:bHLH60*). The *LUC* gene, under control of the 2.6 kb *FT* promoter, was used as the reporter (*ProFT:LUC*) and the *35S* promoter-driven *REN* gene served as the internal control. The negative control effector used was *35S:GFP*. *RGL1* was inserted into the vector pGreenII 62-SK under control of the *35S* promoter to serve as the effector (*35S:RGL1*). When *35S:bHLH48* was co-expressed with *ProFT:LUC*, the LUC activity was significantly increased compared with co-expression of *35S:GFP* and *ProFT:LUC*, indicating that bHLH48 functions as a transcription activator of *FT*. When *35S:RGL1* was co-expressed with *35S:bHLH48* and *ProFT:LUC*, the transcription activation of *ProFT:LUC* was largely repressed. In contrast, *35S:RGL1* did not significantly affect LUC activity when co-expressed with *ProFT:LUC* (Fig. 6B). Similar results were also obtained for bHLH60 (Fig. 6B). These data suggest that RGL1 inactivates the transcriptional regulatory activities of bHLH48 and bHLH60.

### *Overexpression of* bHLH48 *and* bHLH60 *rescues the late-flowering phenotype of* RGL1 *overexpressing plants*

**Fig. 7.**
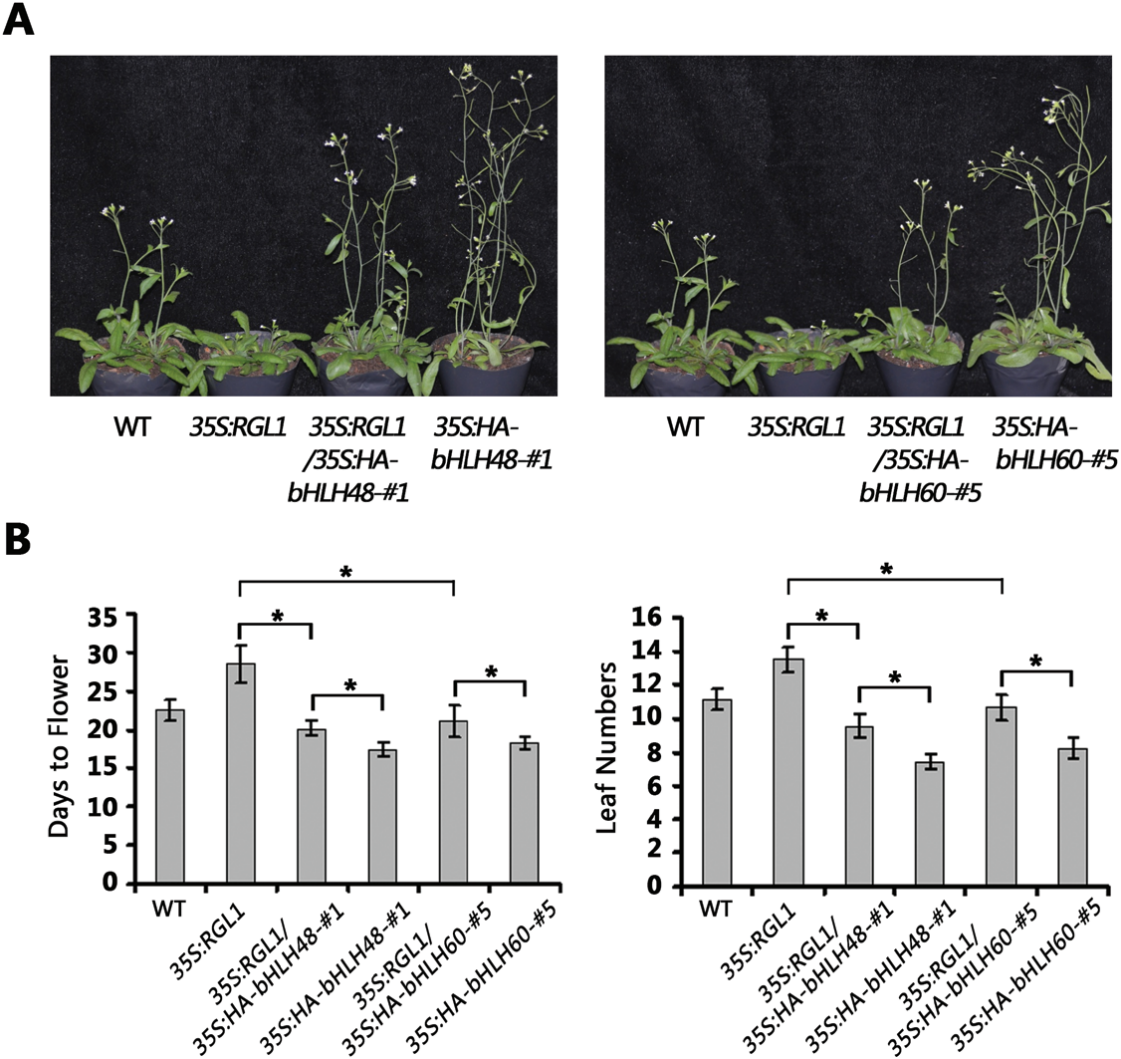
Phenotype rescue of *35S:RGL1* plants by the overexpression of *bHLH48* and *bHLH60*. (A) Flowering phenotype. The plants indicated were grown under LD conditions for 30 days when the pictures were taken. To obtain *35S:RGL1/35S:HA-bHLH48-#1* and *35S:RGL1/35S:HA-bHLH60-#5* plants, *35S:RGL1* was crossed with *35S:HA-bHLH48-#1* and *35S:HA-bHLH60-#5*. (B) Flowering phenotype. The quantitative flowering times were measured as days to flower and the number of rosette leaves at the day floral buds became visible. The mean values (*n*≥20) are shown, with bars indicating standard deviation. Significant differences between two samples are indicated by *, *P* < 0.05.

To investigate whether RGL1 inhibits flowering by suppressing the transcriptional activity of bHLH48 and bHLH60 towards *FT*, genetic experiments were performed. Transgenic plants of *35S:RGL1* were generated and showed a late-flowering phenotype (Wang *et al.*, 2016). Subsequently, *35S:RGL1* were crossed with *35S:HA-bHLH48-#1* and *35S:HA-bHLH60-#5,* respectively. The *35S:RGL1/35S:HA-bHLH48-#1* and *35S:RGL1/35S:HA-bHLH60-#5* plants were identified by PCR and further confirmed by qRT-PCR. When grown under LD conditions, both *35S:RGL1/35S:HA-bHLH48-#1* and *35S:RGL1/35S:HA-bHLH60-#5* showed an early flowering phenotype when compared with *35S:RGL1,* suggesting that overexpression of *bHLH48* or *bHLH60* could suppress the late-flowering phenotype of *35S:RGL1* under LD conditions (Fig. 7A, B). These data suggest that the overexpression of *bHLH48* and *bHLH60* antagonizes the flowering delay caused by *RGL1* overexpression.

## Discussion

GA is an essential hormone that regulates diverse aspects of plant growth and development. Molecular genetic analyses have identified several key factors in the upstream signaling pathway of GA; however, the downstream signaling pathway of GA is still unclear. Recently, several signaling components have been identified as functioning downstream of DELLA proteins, such as PIF3, PIF4, MYC2, GLABRA3 (GL3), and ENHANCER OF GLABRA3 (EGL3) (de Lucas *et al.*, 2008; Feng *et al.*, 2008; Hong *et al.*, 2012; Wild *et al.*, 2012; Qi *et al.*, 2014). Here, bHLH48 and bHLH60 were identified as two new downstream components of the GA signaling pathway.

Although GA accelerates the transition from vegetative growth to flowering, the underlying molecular mechanisms are unclear. A recent study revealed that SPL3 promotes flowering by directly activating the transcription of the *FT* gene in *A. thaliana* (Kim *et al.*, 2012). The proteins encoded by miR156-targeted *SPL* genes, *SPL3*, *SPL4* and *SPL5*, were confirmed to interact with DELLA proteins (Yu *et al.*, 2012). SPL transcription factors promote flowering by activating miR172 and MADS box genes. Meanwhile, SPL proteins can bind to and activate the promoter of *FT*. The opposite flowering phenotypes of *DELLA* and *SPL* overexpressing plants suggest that DELLA proteins negatively affect the functions of SPL proteins. However, when those *SPL* genes are silenced, plants still flower early after GA application, implying that there are other flowering-associated components involved in GA-mediated flowering (Yu *et al.*, 2012). Recently, it was revealed that GA-induced expression of *FT* under LD conditions was dependent on *CO* (Wang *et al.*, 2016). However, the flowering time of the *dellap co-2* double mutant was still earlier than that of the *co-2* single mutant plants, implying that other DELLA-repressed flowering related targets may exist (Wang *et al.*, 2016). This work reveals that bHLH48 and bHLH60 can antagonize the repression effect of RGL1 on flowering.

Gain-of-function of *bHLH48* and *bHLH60* caused an early flowering phenotype (Fig. 2A), whereas their functional repression resulted in a late-flowering phenotype (Fig. 2B), suggesting that bHLH48 and bHLH60 are positive regulators of the flowering transition. In fact, several bHLH proteins have been confirmed to affect flowering positively in *A. thaliana*. Four bHLH proteins, FBH1, FBH2, FBH3 and FBH4, bind preferentially to the E-box *cis*-elements in the *CO* promoter and cause early flowering, regardless of photoperiod (Ito *et al.*, 2012). In addition, CRY2-interacting bHLH 1 (CIB1), CIB2, CIB4 and CIB5 act redundantly to activate the transcription of *FT* in *A. thaliana* (Liu *et al.*, 2008; Liu *et al.*, 2013). The expression of *FT* positively correlated with *bHLH48* and *bHLH60* (Fig. 3A, 3D). ChIP experiments confirmed that bHLH48 associates with the promoter of *FT* (Fig. 4C). These data suggest that *FT* is a direct target of bHLH48 and bHLH60. Liu *et al*. (2014) previously showed that a short proximal promoter and a distal regulatory region, block A and C, respectively, are necessary and sufficient to induce *FT* expression under LD conditions. Our ChIP experiment indicated that bHLH48 was associated with a region between blocks A and C. Recently, WRKY71 and TARGET OF EAT1 (TOE1) were reported to regulate *FT* expression by binding to the region between block A and C (Zhai *et al.*, 2015; Yu *et al.*, 2012). This implies that other regions beyond block A and C also contribute to the expression of *FT*. When *bHLH48* or *bHLH60* was overexpressed in the *ft-11* mutant plants, no early flowering phenotype was observed (Fig. 3E), which suggests that the flowering acceleration in *bHLH48* and *bHLH60* overexpressing plants depends on the upregulation of *FT*. Previous reports demonstrated that GA affects flowering transition by regulating the expression of *LEAFY* and *SUPPRESSOR OF OVEREXPRESSION OF CONSTANS 1* (*SOC1*) (Blázquez *et al.*, 1998; Moon *et al.*, 2003). Investigation of *LEAFY* and *SOC1* indicated that *SOC1,* but not *LEAFY,* was positively regulated by bHLH48 and bHLH60 (Supplementary Fig. S7). Further investigation is required to clarify whether *SOC1* is directly regulated by bHLH48 and bHLH60.

Transcript abundance analysis suggested that both *bHLH48* and *bHLH60* were not affected by exogenous GA application (Fig. 5A, B) and that they did not follow a circadian rhythm pattern similar to that of *FT* (Supplementary Fig. S8). Although *bHLH48* and *bHLH60* were constitutively expressed, the circadian rhythm of *FT* was not affected. This implies that *bHLH48* and *bHLH60* are regulated by GA at the post-transcriptional level. Our recent work revealed that exogenous GA application affects the amplitude of *FT* but not the circadian rhythm of *FT* (Wang *et al.*, 2016). Other groups also found that CIB proteins primarily affect the amplitude but not the circadian rhythm of *FT* in either *CIB* overexpressing plants or *CIB* mutants (Liu *et al.*, 2008; Liu *et al.*, 2013). A similar case was found in the effect of FLOWERING BHLH (FBH) proteins on their target, *CO* (Ito *et al.*, 2012). The overexpression of *bHLH48* and *bHLH60* only caused early flowering under LD conditions but not under SD conditions (Supplementary Fig. S4). This evidence suggests that bHLH48 and bHLH60 regulate flowering specifically under LD conditions. A recent study found that a bHLH transcription factor, NO FLOWERING IN SHORT DAY (NFL), promotes flowering specifically under SD but not under LD conditions (Sharma *et al*., 2016). The bHLH transcription factors therefore have diverged to control flowering under LD and SD conditions in *A. thaliana*.

It was reported that CIB1 and other CIB proteins form heterodimers that have a higher binding affinity than the CIB homodimers to the non-canonical E-box (Liu *et al.*, 2013). The Arabidopsis protoplast transient expression assay indicated that the co-expression of *bHLH48* and *bHLH60* significantly activated the *FT* promoter compared with the expression of *bHLH48* or *bHLH60* alone (Supplementary Fig. S9), suggesting their synergistic role in regulating *FT*. DELLA proteins often suppress the functions of their target proteins. For example, DELLA proteins bind JAZ1 competitively and prevent its inhibitory effect on JA-responsive genes (Hou *et al.*, 2010). DELLA proteins also interact with PIF3 and PIF4 and block their transcriptional activity by binding to their DNA-recognition domains (de Lucas *et al.*, 2008; Feng *et al.*, 2008). Similar DNA-binding inhibitions by DELLA proteins also occur for the other two bHLH transcription factors, MYC2 (Hou *et al*., 2012) and ALC (Arnaud *et al.*, 2010). The ChIP assay suggested that GA promotes the DNA-binding activity of bHLH48 with *FT* (Fig. 5C). Thus, it is likely that RGL1 degradation resulting from GA application releases the DNA-binding domain of bHLH48. Furthermore, the transient expression assays confirmed that RGL1 suppresses the transcription activation of *FT* by bHLH48 and bHLH60. In support of these results, genetic analysis suggested that RGL1 alleviates the early flowering phenotypes of *bHLH48* and *bHLH60* overexpressing plants (Fig. 7B). GA can facilitate the degradation of DELLA proteins. When exogenous GA3 was applied to promote flowering, *bhlh48bhlh60-1* and *bhlh48bhlh60-2* mutants were less insensitive to GA3 compared with wild-type, as shown by the leaf reduction ratio (Supplementary Fig. S10). Collectively, these data are indicative of a positive function of bHLH48 and bHLH60 factors in the GA-mediated flowering pathway. A putative working model was proposed. Under GA limitation conditions, DELLA proteins sequester bHLH48 and bHLH60 into an inactive complex, blocking their binding to the *FT* promoter. In the presence of GA, the DELLA proteins are degraded, which releases the positive regulators bHLH48 and bHLH60 and leads to flowering.

Both *bhlh48* and *bhlh60* single mutants exhibited a flowering time identical to wild-type plants, implying functional redundancy between these two bHLH genes. In fact, the duplication events account for 40% of the bHLH genes, which in turn accounts for high functional redundancy between proteins of the same subgroup (Heim *et al.*, 2003). Transcription factors bHLH48 and bHLH60 belong to the bHLH XII subgroup, which contains 16 members (Supplementary Fig. S2). It is noteworthy that all five CIBs and two CIB LIKE proteins are also contained in this subgroup. Four CIBs have been observed to promote flowering by binding to the *FT* promoter (Liu *et al.*, 2008; Liu *et al.*, 2013). Moreover, the dominant repression of CIBs also causes a late-flowering phenotype (Liu *et al.*, 2013). Thus, it is likely that bHLH48, bHLH60 and the CIBs have high functional redundancy during the regulation of flowering. However, it is unclear whether DELLA proteins also interact with and inhibit the other members in the bHLH XII subgroup to regulate flowering. Further investigation is required to confirm this hypothesis.

## Supplementary data

Supplementary data are available at *JXB* online.

Figure S1. Phenotypes of *bHLH48* and *bHLH60* mutant plants.

Figure S2. Phylogenetic tree of the bHLH subgroup XII.

Figure S3. Real-time PCR analysis of overexpressing plants.

Figure S4. Overexpressing plants grown under SD.

Figure S5. *bhlh48bhlh60* double mutants grown under SD.

Figure S6. Phenotypes of dominant repression lines.

Figure S7. Expression patterns of *LEAFY* and *SOC1* in overexpressing transgenic plants.

Figure S8. Daily expression patterns of *bHLH48* and *bHLH60*.

Figure S9. bHLH48 and bHLH60 synergistically regulate *FT*.

Figure S10. Reduced GA response in *bhlh48bhlh60* mutants.

Supplemental Table 1. Primers used in this study.

## Supplementary Material

supplementary_figures_S1_S10_Table_S1

## Abbreviations:

ChIP: chromatin immunoprecipitation
CIB: CRY2-interacting bHLH
CO: CONSTANS
FT: FLOWERING LOCUS T
GA: gibberellin
GAI: GA INSENSITIVE
GID1: GIBBERELLIN INSENSITIVE DWARF1
LD: long-day
LUC: LUCIFERASE
MS: Murashige and Skoog
PIF: PHYTOCHROME INTERACTING FACTORS
REN: RENILLALUCIFERASE
RGL: REPRESSOR OF GA1-3-LIKE
SD: short-day
SOC: SUPPRESSOR OF OVEREXPRESSION OF CONSTANS
SPL: SQUAMOSA PROMOTER BINDING-LIKE.
