# Supplementary material for: Two DELLA-interacting proteins bHLH48 and bHLH60 regulate flowering under long-day conditions in Arabidopsis thaliana
Source: J Exp Bot. 2017 Jun 7;68(11):2757–67. doi: 10.1093/jxb/erx143 (PMC5853475; doi:10.1093/jxb/erx143)
Supplement: supplementary_figures_S1_S10_Table_S1 [file erx143_suppl_supplementary_figures_s1_s10_table_s1.pdf]

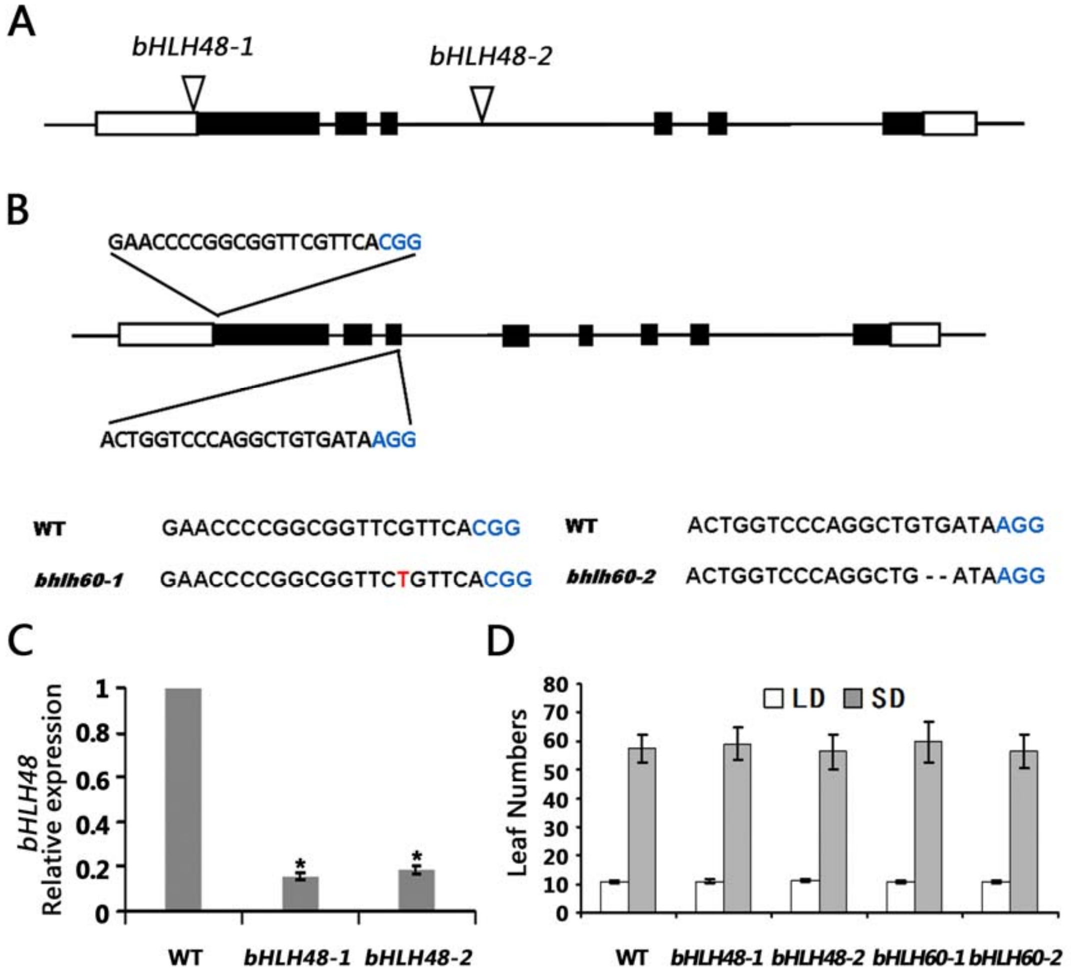

Figure S1. Phenotypes of *bHLH48* and *bHLH60* mutant plants.

(A) The T-DNA insertion position in *bHLH48* mutants.

(B)Gene-targeting sequence of *bHLH60* and the genotypes of the homozygous mutants.

(C)Relative expression of *bHLH48* in the mutants. Significant differences from the wild type are indicated by * ($P < 0.05$).

(D)Plants were grown at 22°C under LD conditions (16-h light/8-h dark) or SD conditions (8-h light/16-h dark). The quantitative flowering times measured as days to flower and the number of rosette leaves at the day floral buds became visible. The mean values (n=20) are shown, with bars indicating standard deviation. Significant difference was indicated by * ($P < 0.05$).

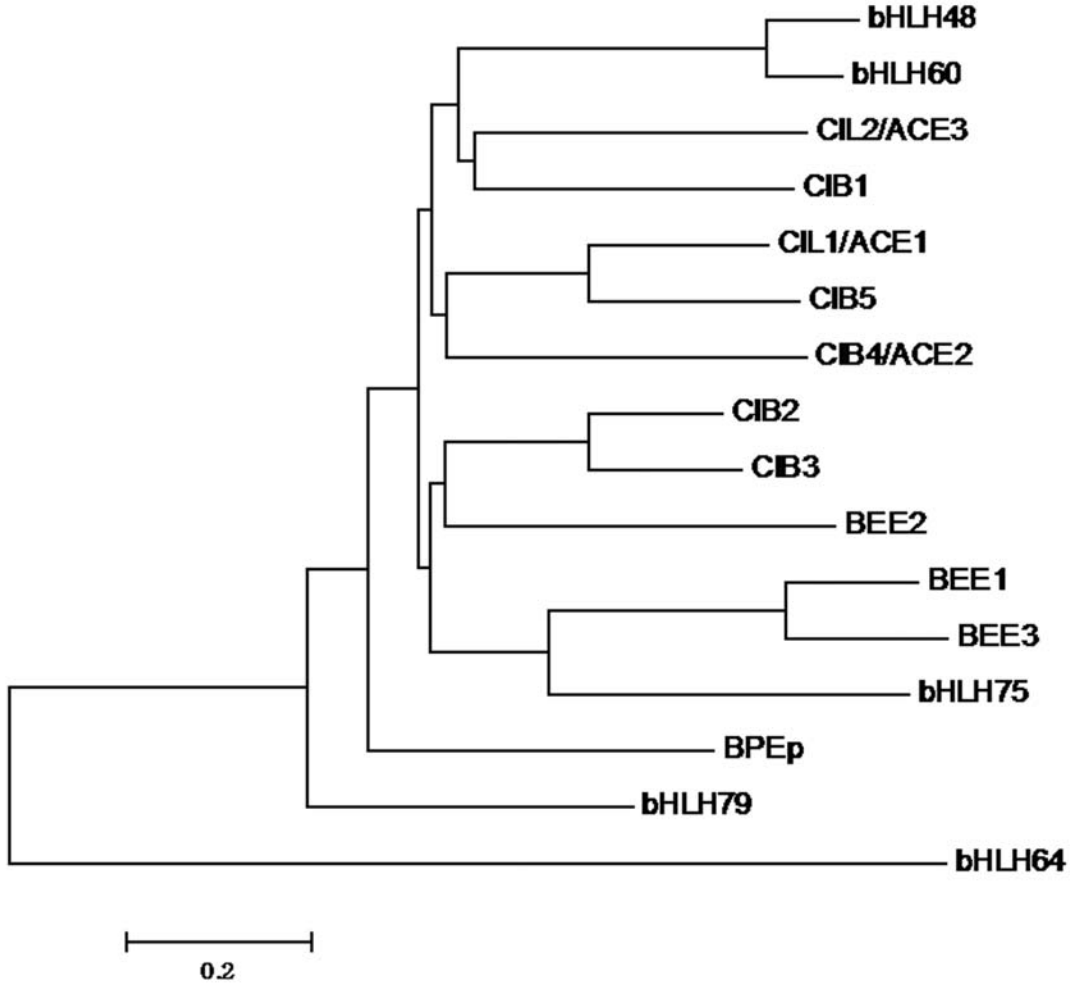

Figure S2. Phylogenetic tree of the bHLH subgroup XII.

The protein sequences were retrieved from TAIR (The Arabidopsis Information Resource). Sequences were aligned by Clustal X1.5. The neighbor-joining phylogenetic tree was constructed by MEGA 3.1. The scale bar indicates substitution per site.

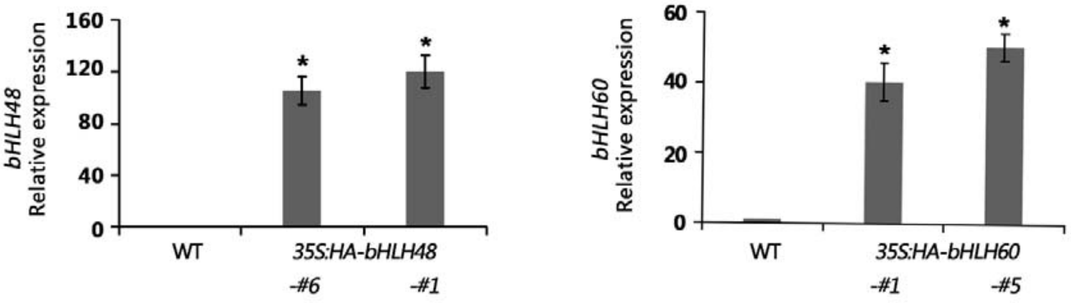

Figure S3. Real-time PCR analysis of overexpressing plants.

Relative expression of *bHLH48* and *bHLH60*. Significant differences from the wild type are indicated by * ($P < 0.05$).

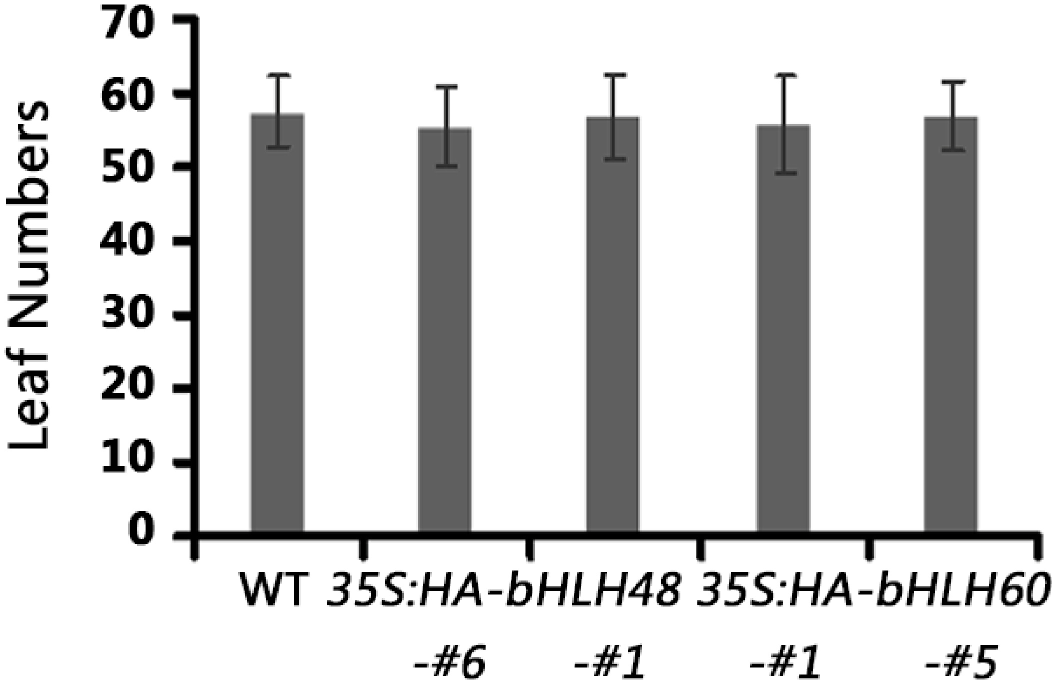

Figure S4. Overexpressing plants grown under SD.

Plants were grown at 22°C under SD conditions (8-h light/16-h dark).The quantitative flowering times measured as the number of rosette leaves at the day floral buds became visible. The mean values (n=20) are shown, with bars indicating standard deviation. Significant difference was indicated by * ($P < 0.05$).

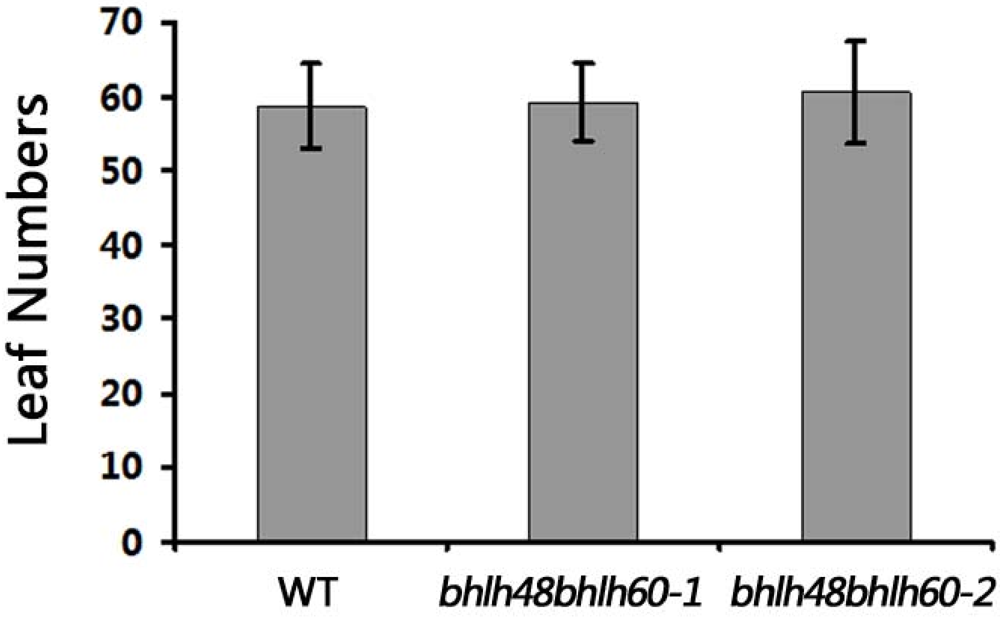

Figure S5. ***bhlh48bhlh60*** **double mutants grown under SD.**

Plants were grown at 22°C under SD conditions (8-h light/16-h dark).The quantitative flowering times measured as the number of rosette leaves at the day floral buds became visible. The mean values (n=20) are shown, with bars indicating standard deviation.

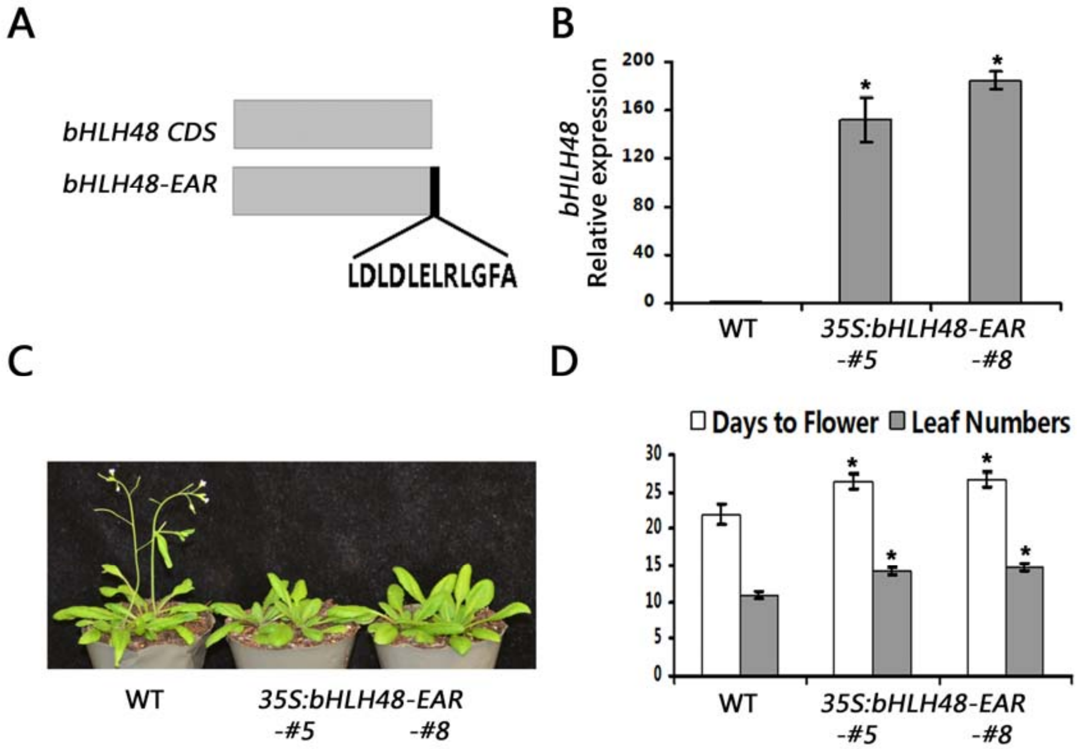

Figure S6. Phenotypes of dominant repression lines.

(A) Construction strategy of dominant repression lines. A twelve-amino acid EAR motif, which serves as a very strong repressor domain, was fused in frame with the 3' end of *bHLH48.*

(B)Relative expression of *bHLH48.* The mean values (n=3) are shown, with bars indicating standard deviation. Significant difference was indicated by * (P < 0.05).

Significant differences from the wild type are indicated by * (P < 0.05).

(C)Flowering phenotype of *35S:bHLH48-EAR* lines. Plants were grown at 22°C under LD conditions (16-h light/8-h dark), 30-day-old plants were shown.

(D)The quantitative flowering times of *35S:bHLH48-EAR* lines. Days to flower

and the number of rosette leaves at the day floral buds became visible. The mean values (n=3) are shown, with bars indicating standard deviation. Significant difference was indicated by * (P < 0.05).

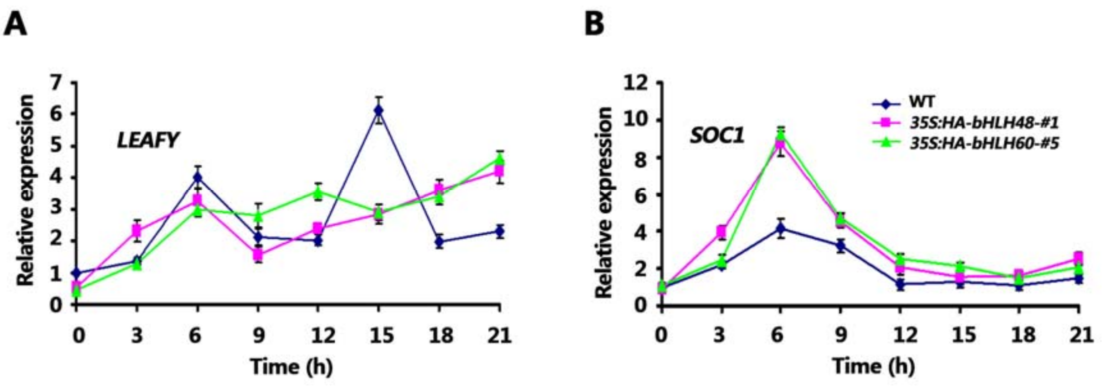

Figure S7. Expression patterns of *LEAFY* and *SOC1* in overexpressing transgenic plants.

(A, B) Daily expression patterns of *LEAFY* and *SOC1* in 8-day-old overexpressing transgenic plants under LD. The mean values (n=3) are shown, with bars indicating standard deviation.

A

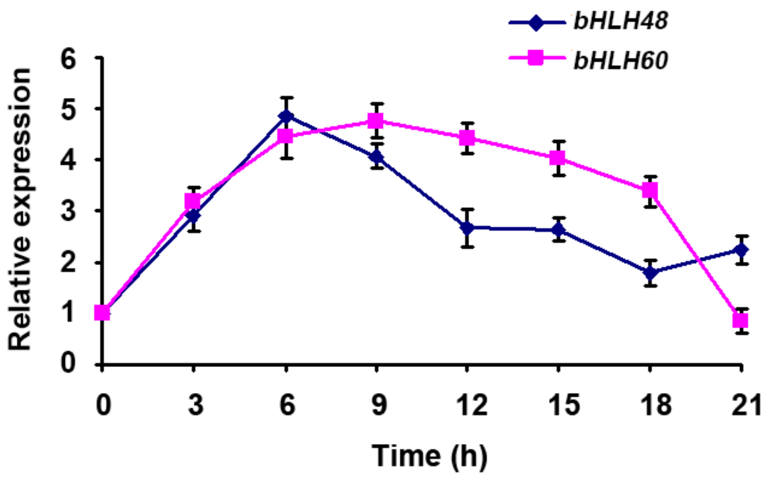

Figure S8. Daily expression patterns of *bHLH48* and *bHLH60*.

(A) RNA levels were measured every 3 hours over a 24-hour cycle in 8-day-old WT under LD conditions. The mean values (n=3) are shown, with bars indicating standard deviation.

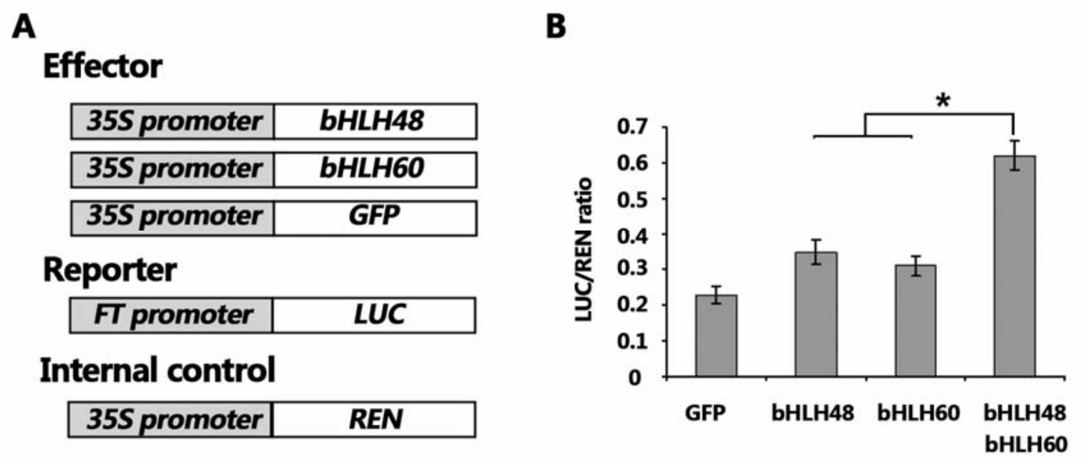

Figure S9. bHLH48 and bHLH60 synergistically regulate *FT*.

(A) Constructs used in transient transactivation assays. *35S:bHLH48*, *35S:bHLH60*, and *35S:GFP* serve as effectors, *ProFT:LUC* as the reporter, and *35S:REN* was the internal control.

(B) The bHLH48/bHLH60 heterodimers have a higher transcription activation of *FT*. The mean values (n=3) are shown, with bars indicating standard deviation. Significant difference was indicated by * (P < 0.05).

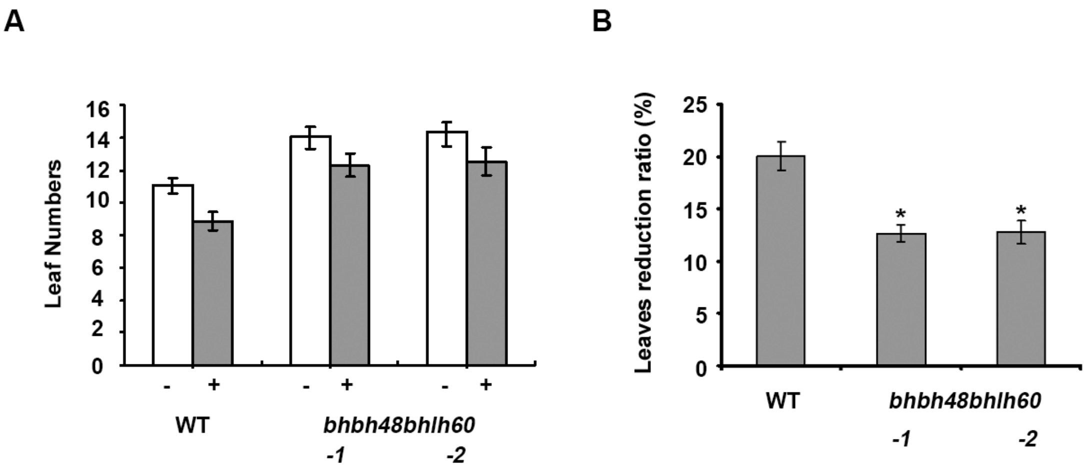

Figure S10. Reduced GA response in *bhlh48bhlh60* mutants.

(A) GA response of wild-type and *bhlh48bhlh60* mutants under LDs. We sprayed 10-day-old seedlings once with 100 μM of GA3 (+) or ethanol (mock, -).

(B) The reduction ratio in response to GA. The reduction ratio was calculated as (number of leaves [mock] - number of leaves [GA3])/ number of leaves [mock]. The mean values (n = 15) are shown, with bars indicating standard deviation. Significant differences from the wild-type are indicated by * ($P < 0.05$).

Supplemental Table 1. Primers used in this study.

Mutant screening	
bhlh48-1-A	TAACTGTCAAAATACTCCCCAACTG
bhlh48-1-B	AAGGGTGAAGAGTGGTAGGAGAAATA
bhlh48-2-A	TATCTCACGGGTCACAACCATTT
bhlh48-2-B	TGTCACTTATCATCATCTCCTTCCA
bHLH60-1-seq-A	CTGACTGGAGGATTTGGAGCTAG
bHLH60-1-seq-B	AGAAATCAACCGCTGAGATGAA
bHLH60-2-seq-A	TTCATCTCAGCGGTTGATTTCT
bHLH60-2-seq-B	CGTTAATCACAAATGAAGACCTAATG
Gene editing	
bHLH60-1-A	TGAACGAACCGCCGGGGTTCTGACCAATGTTGCTCCCTC
bHLH60-1-B	GAACCCCGGCGGTTCGTTCAGTTTTAGAGCTAGAAATAGCAAGT
bHLH60-2-A	TATCACAGCCTGGGACCAGTTGACCAATGTTGCTCCCTC
bHLH60-2-B	ACTGGTCCCAGGCTGTGATAGTTTTAGAGCTAGAAATAGCAAGT
Promoter	
Pro-bHLH48-F	ATAGGATCCCATGTAAGGCAGAGTACAAGCTGC
Pro-bHLH48-R	ATAGAGCTCTAGCCGGGAAAATCACACTTAAAC
Pro-bHLH60-F	ATAGTCGACCCACACCACTCCACTACGAAGAG
Pro-bHLH60-R	ATACCCGGGAAACGGAAACTCACTGGAAATCTT
Overexpression	
bHLH48-HA-F	ATAGGATCCATGTACGACGTACCAGATTACGCTATGGATCTGACCCAAGGTTTCAG
bHLH48-R	ATAGTCGACTTAGAGCTCCATTTTCACCTGATTTG
bHLH48-EAR-R	TATGTCGACTTAAGCGAAACCCAAACGGAGTTCTAGATCCAGATCGAGGAGCTCCATTTTCACCTGATTTG
bHLH60-HA-F	ATAGGATCCATGTACGACGTACCAGATTACGCTATGGATCTGACTGGAGGATTTGG
bHLH60-R	ATAGTCGACTTACAGCTCCATTTTGACCTGATTTG
BiFC	
YN-bHLH48-F	TATATTTAAATATGGATCTGACCCAAGGTTTCA
YN-bHLH48-R	TATGGATCCGAGCTCCATTTTCACCTGATTTG
YN-bHLH60-F	TATGGATCCATGGATCTGACTGGAGGATTTGG
YN-bHLH60-R	TATTCTAGACAGCTCCATTTTGACCTGATTTG
YC-RGL1-F	TATCATATGGTGGTGGTTTTGGATTCTCAAGAAA
YC-RGL1-R	ATAGGATCCTTCCACACGATTGATTCGCCAC
Yeast two-hybrid	
yhRGL1-F	TATCATATGGTGGTGGTTTTGGATTCTCAAGAAA
yhRGL1-R	ATACCCGGGTTATTCCACACGATTGATTCGCCACGC
yhbHLH48-F	TATGAATTCATGGATCTGACCCAAGGTTTCA
yhbHLH48-R	TATGGATCCTTAGAGCTCCATTTTCACCTGATTT
yhbHLH60-F	TATGAATTCATGGATCTGACTGGAGGATTTGG
yhbHLH60-R	TATGGATCCTTACAGCTCCATTTTGACCTGATTTG
Transient expression	
ProFT-LUC-F	ATAAAGCTTAGCTTTATGAGCTTAAACATATGCA
ProFT-LUC-R	ATACCCGGGCTTTGATCTTGAACAAACAGGTGG
bHLH48-SK-F	TATGGATCCATGGATCTGACCCAAGGTTTCA

bHLH48-SK-R	TATGTCGACTTACAGCTCCATTTTGACCTGATTTG
bHLH60-SK-F	TATGGATCCATGGATCTGACTGGAGGATTTGG
bHLH60-SK-R	TATGTCGACTTACAGCTCCATTTTGACCTGATTTG
GFP-SK-F	TATGAGCTCATGAAGAAGAAGAGAAAGCGGAA
GFP-SK-R	TATGGATCCTTACTTGTACAGCTCGTCCATGC
qRT-PCR	
qbHLH48-F	AGGCTGTGATAAGATTCAGGGC
qbHLH48-R	GCCGTTCTCTGAAGCCAGTA
qbHLH60-F	TGTGATAAGATTCAAGGTACCGC
qbHLH60-R	GCAGTTGCCGGTGATGAAAG
qFT-F	CTGGAACAACCTTTGGCAAT
qFT-R	TACACTGTTTGCCTGCCAAG
qCO-F	CTACAACGACAATGGTTCCATTAAC
qCO-R	CAGGGTCAGGTTGTTGCTCTACT
qGI-F	TTCTTCTGCGGGCAACTGAT
qGI-R	TCGACCACTGCTAGTCCAGATG
qACT2-F	TGTGCCAATCTACGAGGGTTT
qACT2-R	TTTCCCGCTCTGCTGTTGT
ChIP	
FTa-F	TAGTTAAACGGCAAAAATCTTCAAT
FTa-R	TCTTTATCTTTCGAAGATGCATTGT
FTb-F	ATGTCAATGCTTACTATATCATCTTACGA
FTb-R	AATATTGGACAGGAGAGCTCAGTACTAT
FTc-F	CTATGGACGTGTAGAACAGTGGCT
FTc-R	AACCATCCATTTGCACGACC
FTd-F	TGACTATTCTCAAATGTCCTGGTTC
FTd-R	CCACCACACTAATACACTGGAAATATT
FTe-F	TGGTTATGATTTCACCGACCC
FTe-R	CCAAGCCATTAGTCACCTCTCTT
FTf-F	AGTTGAGATTGGTGGAGAAGACCT
FTf-R	TGCAGATATTTTAATTAGCAAAAAGG
TUB2-F	ATGGAAACCCAAACATCTTTACC
TUB2-R	TAATATTGTGCACATGTTTGTGGC
